# Time and Eruption Sequence of Permanent Teeth in Hyderabad Children: A Descriptive Cross-sectional Study

**DOI:** 10.5005/jp-journals-10005-1534

**Published:** 2018-08-01

**Authors:** Penmatsa Chaitanya, Jampanapalli S Reddy, Konda Suhasini, Inguva H Chandrika, Dalavai Praveen

**Affiliations:** 1 Reader, Department of Pedodontics and Preventive Dentistry, Vishnu Dental College, Bhimavaram, Andhra Pradesh, India; 2 Professor and Head, Department of Pedodontics and Preventive Dentistry Government Dental College and Hospital, Hyderabad Telangana, India; 3 Associate Professor, Department of Pedodontics and Preventive Dentistry Government Dental College and Hospital, Hyderabad Telangana, India; 4 Assistant Professor, Department of Pedodontics and Preventive Dentistry Government Dental College and Hospital, Hyderabad Telangana, India; 5 Assistant Professor, Department of Conservative Dentistry & Endodontics, Vishnu Dental College, Bhimavaram, Andhra Pradesh, India

**Keywords:** Eruption sequence, Eruption time, Permanent dentition.

## Abstract

**Context:**

Eruption of teeth is influenced by various factors. Very few studies were carried out in the past on the eruption time of teeth in the Indian population.

**Aim:**

The aim of the study is to determine the time and sequence, gender differences, pattern of symmetry in the eruption of permanent teeth in Hyderabad children.

**Materials and methods:**

In this cross-sectional study, 1654 schoolchildren (806 girls and 848 boys) in the age group of 5 to 15 years from Hyderabad were examined for the status of eruption of permanent teeth.

**Statistical analysis used:**

Results were subjected to probit’s regression analysis. The average age at eruption of permanent teeth, excluding third molars, was given as the mean [± standard deviation (SD)] in months for each gender. A table of percentiles of the eruption time was also determined.

**Results:**

Unlike most of the earlier studies which showed that girls are ahead of boys in permanent teeth emergence, no such pattern was observed. No significant difference was found in the eruption of permanent teeth in right and left arches. Eruption of all the mandibular teeth, with the exception of right first premolar (44) in both the genders, was earlier than their maxillary counterparts. There was overall delay in the eruption of permanent teeth and significant delay in the eruption of lower canines in both genders.

**Conclusion:**

The significant delay in the eruption of permanent mandibular canines is relevant for orthodontic treatment planning.

**How to cite this article:** Chaitanya P, Reddy JS, Suhasini K, Chandrika IH, Praveen D. Time and Eruption Sequence of Permanent Teeth in Hyderabad Children: A Descriptive Cross-sectional Study. Int J Clin Pediatr Dent 2018;11(4):330-337.

## INTRODUCTION

The term “eruption” is derived from the Latin word “erumpere” which means to break out. It is defined as the “axial or occlusal movement of a tooth from its developmental position within the jaws to its functional position in the occlusal plane.”^1^

Research on timing and sequence of eruption of permanent teeth was done in different races and ethnic groups across many parts of the world. Studies suggest that Caucasians have a delayed time of eruption when compared with other ethnic groups. In addition, Negroes have earlier eruption than Caucasians. Since the end of the nineteenth century, a trend toward earlier eruption of permanent teeth has been reported in industrialized countries. This is thought to be primarily caused by early puberty, which, in turn, is a result of better nutrition and health care for children.

Various factors influence the permanent teeth eruption. Genetic and hormonal factors, geographical, climatic, racial, gender and ethnic differences, as well as economic status, body constitution, nutrition, fluoride, season of birth, temporal variations, and growth parameters together with infrequent general pathological conditions, such as endocrine pathology, irradiation, and developmental syndromes that exert an influence on eruption patterns. Relationship between time of eruption, height, and weight of children was also reported. Children who are below average weight and height for a specific age show a later eruption time than those children who are within the standard range.^2^

The timing of permanent teeth eruption is of considerable importance in the planning of diagnostic, preventive, and therapeutic measures.^3^ As there is limited information on the timing and sequence of permanent teeth eruption in Hyderabad population, this epidemiological survey was conducted.

## MATERIALS AND METHODS

A group of 1654 schoolchildren (806 girls and 848 boys) from kindergarten, primary, and high school ranging in age from 5 to 15 years, from private schools in Kukatpally, Hyderabad, were examined for the study. Wide range of age was chosen in order to include the permanent dentition completely. No selection was made concerning differences in physical or mental development, social status, religion, ethnicity, or whether the child is born in Hyderabad or not.

Initially, before starting, introductory letters were sent to both the schools. Permission was obtained from the school authorities to conduct the study while the parents and subjects were informed verbally about the survey.

### Methodology

Initially, basic information, i.e., age and gender of child, frequency of tooth brushing, occupation of the parent, and address, was recorded. Date of birth was recorded from the school register. The age was calculated from each child’s date of birth to the date of examination and rounded off to the full month.

Inclusion criteria were all normal children and the exclusion criteria were those with systemic diseases, those undergoing orthodontic treatment, premature loss or extraction of any permanent teeth, severe crowding delaying tooth eruption, children with localized pathology or anomaly, congenitally missing teeth, and presence of third molars.

Dental examination was then carried out with the subject seated on an ordinary chair, under natural light and using a mouth mirror and a blunt probe. The teeth were identified according to the two-digit system of the Federation Dentaire Internationale (FDI).

Status of eruption of each permanent tooth was recorded. Even if a small part of the crown is visible clinically penetrating the oral mucous membrane, it is regarded as erupted and recorded accordingly.

### Statistical Analysis

Following the acquisition of data, results were tabulated and the findings were then transferred to computer using standard software (Microsoft Access, version 2000). The calculations were performed according to earlier studies on tooth emergence.

The data were then subjected to “probit analysis” using the numerical approximation of a maximum-likelihood estimation. This method is internationally well established and is repeatedly applied for the estimation of tooth eruption. This method provides reliable results even in small-sized samples.

Probit analysis was used to determine 3rd, 10th, 25th, 50th, 70th, 90th, 95th, and 97th percentiles of eruption for each individual tooth. The mean age of eruption of each tooth was determined and later, the sequence of tooth eruption was determined by referring to the mean age of eruption of individual tooth.

## RESULTS

Table 1 shows the distribution of the sample according to age and gender. The sample was divided into 10 age groups with 12 months (1 year) time interval. Based on the mean time of eruption (Table 2), the sequence of eruption of permanent teeth is as follows:

Maxilla: 6-1-2-4-3-5-7

Mandible: 6-1-2-3-4-5-7

The mean age of eruption of all teeth on right side was earlier in the upper arch except second molar and in the lower arch, the eruption of permanent central incisor, lateral incisor, and second premolar was earlier in the right side (Table 3).

Tables 4 and 5 show different percentiles (P3, P10, P25, P50, P75, P90, P95, and P97) for the time of eruption of the 28 permanent teeth for girls and boys. In 3% of girls examined, 11 had erupted by the age of 94.3 months, and by the age of 108.2 months, 97% of girls had their permanent central incisors (11, 21, 31, and 41). By the age of 97 months, the first four permanent molars had erupted in 97% of girls. Eruption of the four central incisors in boys occurred slightly earlier. By the age of 107.7 months, 97% of boys examined had all their central incisors. Only 3% of boys had all first permanent molars erupted by the age of 80.2 months; 97% of them had all first molars erupted (between the ages of 74.2 and 98.6 months) by the age of 98.6 months. By 149.7 months, all the first premolars were erupted in 97% of girls while in boys, earlier eruption of first premolars was observed. By 149.1 months, all the first premolars were erupted in 97% of boys. By 155.9 months, all the second premolars were erupted in 97% of girls. There was delayed eruption of second premolars in boys compared with girls. By 156.3 months, all the second premolars erupted in 97% of the boys. In 97% of girls, canine erupted by the age of 153 months, while in 97% of boys, canine erupted by the age of 154.4 months, indicating delayed eruption of permanent canines in boys.

**Table 1:** **Table 1:** Distribution of sample according to age and gender

*Age group (months)*		*Girls*		*Boys*		*Total*	
60-71		97		91		188	
72-83		59		77		136.0	
84-95		85		78.0		163	
96-107		92		101		193	
108-119		77		92		169	
120-131		73		83.0		156	
132-143		95		84		179	
144-155		82		62		144	
156-167		54		74		128	
168-180		92		106		198	
Total		806		848		1654	

**Table 2:** **Table 2:** Mean age and SD, minimum and maximum values of permanent teeth eruption (in months) among girls and boys

		*Mean age (months)*				*SD*				*Minimum*				*Maximum*			
Tooth*		*Girls*		*Boys*		*Girls*		*Boys*		*Girls*		*Boys*		*Girls*		*Boys*	
11		101.8		101.5		3.8		4.2		73.9		72.3		108.1		108.2	
12		115.0		115.5		4.1		3.9		85.5		75.1		121.3		121.4	
13		146.5		145.9		4.2		3.9		102.4		114.9		153.3		152.9	
14		139.9		140.1		4.1		4.2		90.2		93.7		147.6		147.6	
15		148.7		148.1		3.7		3.8		111.8		102.6		155.8		155.9	
16		87.8		87.9		4.6		4.7		67.9		61.6		95.2		95.4	
17		161.4		161.3		3.6		3.5		118.4		118.3		166.9		167.4	
21		102.0		101.8		3.7		4.0		67.3		73.8		108.3		108.2	
22		116.2		116.5		4.5		4.2		85.5		75.1		123.4		123.4	
23		147.4		146.7		3.9		4.0		96.3		104.0		154.1		154.5	
24		141.8		142.1		4.7		4.4		95.5		93.7		150.0		150.1	
25		148.8		148.2		3.8		4.2		99.3		102.6		156.3		156.3	
26		87.9		88.4		5.7		6.3		68.8		61.6		98.1		98.9	
27		160.5		160.6		3.7		3.4		118.4		123.6		166.0		165.8	
31		89.4		90.1		5.1		5.5		66.9		61.6		99.1		99.1	
32		104.7		104.5		3.9		4.4		69.2		68.4		112.5		112.6	
33		140.3		140.8		3.9		3.8		100.1		98.2		147.6		147.6	
34		141.5		141.9		3.9		4.1		104.4		84.3		148.9		148.9	
35		148.7		148.1		3.7		3.8		111.2		98.2		155.8		155.9	
36		83.9		82.6		5.1		5.1		67.3		61.6		91.5		91.6	
37		153.2		153.6		3.9		4.2		118.4		119.8		160.5		160.2	
41		88.8		89.0		5.1		5.3		66.9		61.6		98.1		98.2	
42		104.4		104.2		3.6		4.1		69.2		73.8		111.8		111.8	
43		141.2		141.4		3.7		3.8		103.5		104.4		148.0		148.1	
44		142.1		142.4		4.1		4.1		96.3		84.3		149.5		149.3	
45		148.6		147.8		3.6		3.6		102.7		98.2		155.1		154.6	
46		84.9		83.4		5.2		5.4		67.3		61.6		92.8		92.8	
47		153.8		154.8		3.8		3.8		118.0		129.0		160.7		160.8	

*The FDI tooth notations are used

**Table 3:** **Table 3:** Comparison of mean age (in months) of permanent teeth eruption between contralateral sides of maxillary and mandibular arches among girls and boys

		*Right side (mean age ± SD)*				*Left side (mean age ± SD)*			
*Maxillary arch*		*Girls*		*Boys*		*Girls*		*Boys*	
Central incisor		101.8 ± 3.8		101.5 ± 4.2		102.0 ± 3.7		101.8 ± 4.0	
Lateral incisor		115.0 ± 4.1		115.5 ± 3.9		116.2 ± 4.5		116.5 ± 4.2	
Canine		146.5 ± 4.2		145.9 ± 3.9		147.4 ± 3.9		146.7 ± 4.0	
First premolar		139.9 ± 4.1		140.1 ± 4.2		141.8 ± 4.7		142.1 ± 4.4	
Second premolar		148.7 ± 3.7		148.1 ± 3.8		148.8 ± 3.8		148.2 ± 4.2	
First molar		87.8 ± 4.6		87.9 ± 4.7		87.9 ± 5.7		88.4 ± 6.3	
Second molar		161.4 ± 3.6		161.3 ± 3.5		160.5 ± 3.7		160.6 ± 3.4	
		*Right side (mean age ± SD)*				*Left side (mean age ± SD)*			
*Mandibular arch*		*Girls*		*Boys*		*Girls*		*Boys*	
Central incisor		88.8 ± 5.1		89.0 ± 5.3		89.4 ± 5.1		90.1 ± 5.5	
Lateral incisor		104.4 ± 3.6		104.2 ± 4.1		104.7 ± 3.9		104.5 ± 4.4	
Canine		141.2 ± 3.7		141.4 ± 3.8		140.3 ± 3.9		140.8 ± 3.8	
First premolar		142.1 ± 4.1		142.4 ± 4.1		141.5 ± 3.9		141.9 ± 4.1	
Second premolar		148.6 ± 3.6		147.8 ± 3.6		148.7 ± 3.7		148.1 ± 3.8	
First molar		84.9 ± 5.2		83.4 ± 5.4		83.9 ± 5.1		82.6 ± 5.1	
Second molar		153.8 ± 3.8		154.8 ± 3.8		153.2 ± 3.9		153.6 ± 4.2	

In 97% of girls, second molars erupted by the age of 166.8 months, while in 97% of boys, second molars erupted by the age of 167.2 months, indicating delayed eruption of second molars in boys.

**Table 4:** **Table 4:** Different percentiles of eruption time (in months) of permanent teeth in girls

Tooth*		*P 3*		*P 10*		*P 25*		*P 50*		*P 75*		*P 90*		*P 95*		*P 97*	
11		94.3		95.7		99.2		102.2		104.9		106.5		107.2		107.6	
12		108		108.7		111.5		115.3		118.4		120.3		121		121.2	
13		139.8		140.5		142.8		146.5		149.9		152.1		152.9		153	
14		133.9		134.7		136.7		139.5		144.1		146.2		147.1		147.2	
15		142.3		144.1		145.4		149		151.9		153.6		154.9		155.3	
16		80.2		81.1		83.4		88.3		92		93.7		94.8		95.1	
17		154.9		155.8		158.6		161.4		164.8		166		166.6		166.8	
21		94.8		95.8		99.3		102.6		105.1		107		107.7		108.2	
22		108.3		109.7		112.4		117.1		120.1		121.8		123		123.2	
23		140.9		141.3		144.3		147.5		150.7		152.7		153.1		153.6	
24		134.8		135.5		137.8		141.1		146.2		149.1		149.6		149.7	
25		142.3		144.1		145.4		149.3		152		154.1		155.4		155.9	
26		78		80.2		83		88.5		92.7		95.3		96.4		97	
27		153.8		155		158.3		160.5		164.1		165.3		165.5		165.8	
31		81.1		81.8		85.4		90.2		93.3		96.4		98		99	
32		98.5		99.6		101.7		104.4		108		110.3		111.8		112.2	
33		134.6		135.1		137.1		140		144.2		146.3		147.1		147.3	
34		135.4		136.6		138		140.9		144.9		147.3		148		148.6	
35		142.3		144.1		145.4		149		151.9		153.6		154.9		155.3	
36		74.2		76.6		80.2		84.1		88.5		90.9		91.3		91.4	
37		147.6		148.7		149.7		152.4		156.5		159.1		159.7		160.2	
41		80.4		81.4		84.3		89.8		92.9		95.7		96.6		97.1	
42		98.3		99.5		101.5		104.3		107.1		109.9		110.5		111.3	
43		135.4		136.6		138		140.8		144.7		146.8		147.4		147.8	
44		136		137		138.4		141.2		145.5		148.1		149.2		149.4	
45		142.3		144.1		145.4		148.9		151.7		153.1		154.5		155	
46		75.1		77.1		81		85.5		90.1		91.4		92.3		92.6	
47		148.8		149.4		150.5		152.9		157.7		159.3		160.1		160.5	

*The FDI tooth notations are used.

**Table 5:** **Table 5:** Different percentiles of eruption time (in months) of permanent teeth in boys

Tooth*		*P 3*		*P 10*		*P 25*		*P 50*		*P 75*		*P 90*		*P 95*		*P 97*	
11		93.9		95.1		98.3		101.3		105.3		106.9		107.5		107.7	
12		107.9		110.3		112.5		116.5		118.7		120.4		120.8		121.3	
13		139.7		140.5		142.5		145.7		148.8		152		152.6		152.8	
14		133.7		134.3		136.6		139.7		144		146		147.3		147.5	
15		142.2		143		144.7		147.9		151.6		153.6		154.5		155.5	
16		80.1		81.4		83.7		88		92.3		94		94.8		94.9	
17		154.5		156.3		158.2		161.4		164.3		165.7		167		167.2	
21		94.8		96.1		98.6		101.8		105.8		107		107.5		107.7	
22		109.1		110.6		112.8		117.1		119.8		122.1		123.1		123.2	
23		140.4		141.2		143.5		146.9		149.2		152.6		153.7		154.4	
24		135		136		138.4		141.7		145.8		148.3		149		149.1	
25		141.7		142.8		144.5		147.9		151.9		154.5		156		156.3	
26		77.6		79.4		82.8		88.7		93.8		96.9		98.3		98.6	
27		154.3		155.9		157.9		161		163.6		165.2		165.7		165.7	
31		81		82.4		85.1		90.5		94.7		97.4		98.6		98.8	
32		97.4		98.8		100.5		104.8		107.5		111.1		111.8		112.1	
33		134.8		135.8		137.9		140.4		144.3		146.4		147.3		147.5	
34		135.6		136.5		138.5		141.7		145.4		147.9		148.3		148.7	
35		142.2		143		144.7		147.9		151.6		153.6		154.5		155.5	
36		74.7		75.7		77.7		82.6		86.6		89.7		90.9		91.3	
37		147.5		148		149.1		152.9		157.7		159.4		160		160.2	
41		80.3		81.8		84.3		89.5		93.5		96.3		97.1		97.4	
42		97.4		98.7		100.3		104.4		107.1		110.5		111.1		111.3	
43		135.5		136.3		138.3		141		144.5		147		147.5		147.9	
44		135.8		136.7		139		142		145.9		148.3		148.9		149.1	
45		142.2		142.9		144.6		147.6		151.3		152.8		154.4		154.5	
46		74.7		76.1		78.4		83.2		88		91.2		92.2		92.2	
47		148.6		149.1		151.8		155.7		158.1		159.8		160.2		160.7	

*The FDI tooth notations are used.

**Table 6:** **Table 6:** Mean age (in years) of permanent teeth eruption among girls and boys compared with standard time of eruption (in years)

Tooth*		*Standard time of eruption (in years)*		*Mean age of eruption in girls (in years)*		*Mean age of eruption in boys (in years)*	
11		7-8		8.48		8.45	
12		8-9		9.58		9.62	
13		11-12		12.20		12.15	
14		10-11		11.65		11.67	
15		10-12		12.39		12.34	
16		6-7		7.31		7.32	
17		12-13		13.34		13.44	
21		7-8		8.5		8.48	
22		8-9		9.68		9.70	
23		11-12		12.28		12.22	
24		10-11		11.81		11.84	
25		10-12		12.4		12.35	
26		6-7		7.32		7.36	
27		12-13		13.37		13.38	
31		6-7		7.45		7.50	
32		7-8		8.72		8.70	
33		9-10		11.69**		11.73**	
34		10-12		11.79		11.82	
35		11-12		12.39		12.34	
36		6-7		6.99		6.88	
37		11-13		12.76		12.8	
41		6-7		7.4		7.41	
42		7-8		8.7		8.68	
43		9-10		11.76**		11.78**	
44		10-12		11.84		11.86	
45		11-12		12.38		12.31	
46		6-7		7.07		6.95	
47		11-13		12.81		12.90	

*The FDI tooth notations are used; **Indicates delayed eruption compared with standard eruption time given by Logan and Kronfeld4^4^

Except the lower left and right first premolar teeth (34, 44) and left and right lower second permanent molar teeth (37, 47), all the other maxillary and mandibular permanent teeth had erupted beyond the range of standard eruption time.^4^ But this difference was not statistically significant (Table 6).

**Graph 1:**
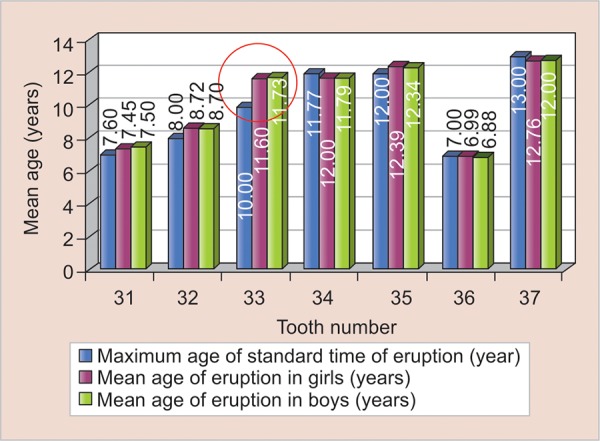
Mean age of eruption of left mandibular teeth among girls and boys compared with standard time of eruption

**Graph 2:**
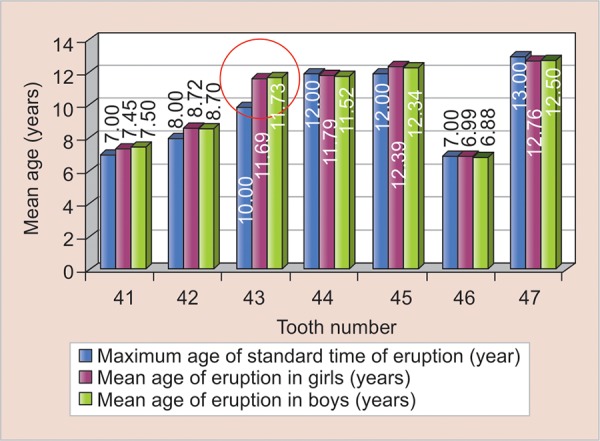
Mean age of eruption of right mandibular teeth among girls and boys compared with standard time of eruption

On comparison with the standard eruption time, there was considerable delay in the eruption of lower left and right canines (33 and 34) in both girls and boys. The standard time of eruption of lower canines is 9 to 10 years, while the mean age of eruption of 33 in girls was 11.69 years and in boys, it was 11.73 years. Similarly, the mean age of eruption 43 in girls was 11.76 years, and in boys, it was 11.78 years (Graphs 1 and 2).

Based on the above tables, the sequence of eruption of maxillary and mandibular teeth is distinct. No difference in the sequence of eruption is observed between girls and boys.

## DISCUSSION

Indians form one-sixth of the global population and they differ from the Western population racially, genetically, and environmentally. So, the standard eruption age based on Western population cannot be applied to the Indian population.

Very few studies were carried out in the past on the eruption time of teeth in the Indian population.^5-8^ The studies done by Tendon^9^ and Gunashekhar and Tenny^8^ showed differences in the eruption of deciduous dentition, and the study done by Gupta et al^10^ showed differences in the eruption of permanent dentition in a south Indian population as compared with the data given by Logan and Kronfeld^4^ that was based on Western population. These findings strongly suggest that racial differences between the two populations is the main factor underlying the variations in eruption timings.^5^

Most of the earlier studies done in children from Tanzania,^1^ Pima India,^11^ Kingston (New York),^12^ West Africa (Gambia),^13^ Japan,^14^ Danish,^15^ Hawaii,^16^ Australia,^17^ French,^18^ Kelatan (North-eastern Malaysia),^19^ Izmir (Turkey),^20^ Malaysia,^21^ Flemish,^22^ Patiala District (India),^23^ and Kerman province^24^ confirm that the girls were ahead of boys in permanent tooth emergence. Unlike these studies, no such pattern was observed in the present study. In the anterior teeth, eruption of upper lateral incisors, lower central incisors, and lower canines was earlier in girls and among posterior teeth, eruption of upper and lower first premolars, upper first molar, and with the exception of upper right second molar, eruption of second molars was earlier in girls. Eruption of all the remaining teeth was earlier in boys. Garcia-Godoy et al^25^ reported early eruption of permanent teeth in girls with exception of canine and second premolar in maxilla and first premolar in mandible in South Eastern Dominican children. In a study done in North Ireland,^26^ earlier eruption of permanent teeth was found in girls with exception of permanent second molar eruption in both the arches. In a study done in Iran,^2^ eruption of permanent teeth was earlier in girls with exception of maxillary second premolar. Similar results were reported in Zagreb children.^27^ The study done by Gunashekar and Tenny^8^ in Indian children showed that the boys showed earlier eruption of all the primary teeth except maxillary second molar and maxillary/mandibular first molars which erupted earlier in girls.

The differences between the emergence ages of contralateral teeth were very small, confirming earlier studies done in Tanzania,^1^ Pima Indians,^11^ Japan,^14^ Danish children,^15^ Australia,^17^ French children,^18^ North Eastern Malaysia,^19^ Jatsikh chidren,^23^ Kerman province,^24^ Northern Ireland,^26^ and Athens (Greece).^28^ Significant differences in the eruption of contra lateral permanent teeth were reported in Iran,^2^ Flemish,^22^ and Zagreb children.^27^ In Spanish children,^29^ a slight earlier emergence of teeth was noted on the left side and with a tendency toward early eruption in boys.

Previous studies done in children from Tanzania,^1^ Tehran (Iran),^2^ Australia,^17^ and Flemish^30^ reported earlier eruption of mandibular teeth before the eruption of their maxillary counterparts. In this study, eruption of all the mandibular teeth, with the exception of right first premolar in both the genders, was earlier than their maxillary counterparts. This difference in the premolar eruption confirmed the results of earlier studies done in Pima Indian,^11^ New York,^12^ West Africa,^13^ French,^18^ North Eastern Malaysia,^19^ Turkey,^20^ North Ireland,^26^ Zagreb,^27^ and Greece^28^ children.

Similar emergence ages for antagonist first molars were observed in the study done by Knott and Meredith^31^ and Krumholt et al.^32^ Most researchers reported that mandibular first molars emerged at a younger age than their antagonists, while Stones et al,^33^ and Nanda^34^ stated the contrary.

The sequence of eruption in this study was similar to that reported in other studies done in Gambia,^13^ Australia,^17^ China,^7^ Japan,^35^ Kenyans of Asian origin,^36^ and Indian studies from Chennai and Lahore,^37^ Chandigarh,^38^ and Delhi.^39^ On comparison with the earlier studies done on children from Tanzania,^1^ Iran,^2^ Pima India,^11^ New York,^12^ West Africa (Gambia),^13^ Japan,^14^ Danish,^15^ Australia,^17^ French,^18^ Kelatan (NorthEastern Malaysia),^19^ Izmir (Turkey),^20^ Malaysia,^21^ Flemish,^22^ Dominican Republic,^25^ Athens (Greece),^28^ Eskimo,^40^ Finn,^41^ South India,^10^ Nigeria,^42^ and Romania,^43^ there was overall delay in the eruption of permanent teeth. When compared with the age of eruption of permanent canine and premolar teeth in Kerman Province children,^24^ there was early eruption of these teeth in the present study.

It is difficult to explain why there is overall delay in the eruption of permanent teeth on comparison with standard eruption time.^4^ The study done by Gunashekhar and Tenny^8^ showed delayed eruption of primary teeth in Indian children when compared with their counterparts in other populations. A genetic influence could be reason for this emergence pattern. Further studies should analyze this aspect.

The statistically significant delay in the eruption of lower canines corresponds to the results of Kerman Province children.^24^ This delay has an important clinical correlation relative to the development of a normal occlusion and the potential for aberrant development into a malocclusion. Due to very short transition of this period in both the genders in the present study (girls: 0.9 months on right side; 1.2 months on left side, and in boys: 1 month on right side; 1.1 months on the left side), there is substantial opportunity for developing a maloc-clusion. The delay in eruption of lower canines is relevant for orthodontic treatment planning, especially in cases of deficit of space in the canine region. The threat of a dystrophic eruption of canine is eminent in an eruption sequence with the canine following premolars when the first molar emerges mesially.

## SUMMARY AND CONCLUSION

The results of the present study provide an insight into variability in emergence sequence of permanent teeth in children living in Hyderabad. The mean eruption time of the permanent teeth was delayed compared with those presented in earlier published reports. There are several possible reasons for these differences that may relate to either, or both, methodological and biological issues. But this difference was not statistically significant except with the eruption of lower permanent canines.

However, data from the present study cannot be generalized to all the Hyderabad children, as factors related to timing of permanent tooth emergence may vary considerably. The time and sequence of eruption may alter with time and with changes in social status, patterns of health, and health care. Further investigations involving a large sample from the different regions of the city could provide additional insight into tooth emergence trends in Hyderabad. The longitudinal study design will make it possible to further explore the impact of possible disturbing factors on tooth emergence, such as caries and early loss of primary teeth on the emergence of permanent teeth.

## References

[1] Mugonzibwa EA, Kuijpers-Jagtman AM, Laine-Alava MT, Van’t Hoff MA (2002). Emergence of permanent teeth in Tanzanian children. Community Dent Oral Epidemiol.

[2] Moslemi M (2004). An epidemiological survey on time and sequence of eruption of permanent teeth in 4-15-year-olds in Tehran, Iran. Int J Paediatr Dent.

[3] Ekstrand KR, Christiansen J, Christiansen ME (2003). Time and duration of eruption of first and second permanent molars: a longitudinal investigation. Community Dent Oral Epidemiol.

[4] Logan WH, Kronfeld R (1933). Development of the human jaws and surrounding structures from birth to age fifteen. J Am Dent Assoc.

[5] Schour I, Massler M (1941). Development of human dentition. J Am Dent Assoc.

[6] Kaul SS, Pathak RK (1992). Santosh Emergence of deciduous teeth in Punjabi children, North India. Z Morphol Anthropol.

[7] Agarwal KN, Gupta R, Faridi MM, Kalra N (2004). Permanent dentition in Delhi boys of age 5-14 years. Indian Pediatr.

[8] Gunashekhar M, Tenny J (2010). Longitudinal study of age and order of eruption of primary teeth in Indian children. J Clin Exp Dent.

[9] Tendon S (2001). Textbook of pedodontics..

[10] Gupta R, Sivapathasundharam B, Einstein A (2007). Eruption age of permanent mandibular first molar and central incisors in the south Indian population. Indian J Dent Res.

[11] Dahlberg AA, Menegaz-Bock RM (1958). Emergence of the permanent teeth in Pima Indian Children. J Dent Res.

[12] Carlos JP, Gittelsohn AM (1965). Longitudinal studies of the natural history of caries I. Eruption patterns of the permanent teeth. J Dent Res.

[13] Billewicz WZ, Mc Gregor IA (1975). Eruption of permanent teeth in West African (Gambian) children in relation to age, sex and physique. Ann Hum Biol.

[14] Hoffding J, Marda M, Yamaguchi K, Tsuji H, Kuwabara S, Nohara Y, Yoshida S (1984). Emergence of permanent teeth and onset of dental stages in Japanese children. Community Dent Oral Epidemol.

[15] Parner ET, Heidmann JM, Vaeth M, Poulsen S (2001). A longitudinal study of time trends in the eruption of permanent teeth in Danish children. Arch Oral Biol.

[16] Greer MH, Kevin JL (2003). Quantitative evaluation of variance in secondary dentition eruption among ethnic groups in Hawaii. Pacific Health Dialog.

[17] Diamanti J, Townsend GC (2003). New standards for permanent tooth emergence in Australian children. Aust Dent J.

[18] Rousset MM, Boualam N, Delfosse C, Roberts WE (2003). Emergence of permanent teeth: secular trends and variance in a modern sample. J Dent Child.

[19] Nizam A, Naing L, Mokhtar N (2003). Age and sequence of eruption of permanent teeth in Kelantan, North-eastern Malaysia. Clin Oral Invest.

[20] Wedl JS, Schoder V, Blake FA, Schmelzle R, Friedrich RE (2004). Eruption times of Permanent Teeth in Teenage Boys and Girls in Izmir (Turkey). J Clin Forensic Med.

[21] Hussin AS, Mokhtar N, Naing L, Taylor JA, Mahmood Z (2007). The timing and sequence of emergence of permanent teeth in Malay school children in Kota Bharu, Malaysia. Arch Orofac Sci.

[22] Leroy R, Cecere S, Lesaffre E, Declerck D (2008). Variability in permanent tooth emergence sequences in Flemish children. Eur J Oral Sci.

[23] Kaur I, Singal P, Bhatnagar DP (2010). Timing of permanent teeth emergence and dental caries among Jatsikh children of public and government schools of Patiala district. Anthropologist.

[24] Elham F, Adhamy S (2010). Age and sequence of permanent canine and premolar teeth eruption in 102-174 months old children in Kerman province. Curr Res Dent.

[25] Garcia-Godoy F, Diaz AN, Del Valle JM, Arana EJ (1982). Timing of permanent tooth emergence in a southeastern Dominican schoolchildren population sample. Community Dent Oral Epidemiol.

[26] Kochhar R, Richardson A (1998). The chronology and sequence of eruption of human permanent teeth in Northern Ireland. Int J Paediatr Dent.

[27] Rajic Z, Mestrovic SR, Verzak Z (2000). Chronology, dynamics and period of permanent tooth eruption in Zagreb children (Part II). Coll Antropol.

[28] Wedl JS, Danias S, Schmelzle R, Friedrich RE (2005). Eruption times of permanent teeth in children and adolescents in Athens (Greece). Clin Oral Invest.

[29] Kodali VR (1998). The interface of nutrition and dentition. Indian J Pediatr.

[30] Leroy R, Bogaerts K, Lasaffre E, Declerck D (2003). The emergence of permanent teeth in Flemish children. Community Dent Oral Epidemiol.

[31] Knott VB, Meredith HV (1966). Statistics on eruption of the permanent dentition from serial data for North American white children. Angle Orthod.

[32] Krumholt L, Roed-Petersen B, Bindborg JJ (1971). Eruption times of the permanent teeth in 622 Ugandan children. Arch Oral Biol.

[33] Stones HH, Lawton FE, Bransby ER, Hartley HO (1951). Time of eruption of permanent teeth and time of shedding of deciduous teeth. Br Dent J.

[34] Nanda RS (1960). Eruption of human teeth. Am J Orthod.

[35] Eveleth PB, de Freites JA (1969). Tooth eruption and menarche of Brazilian born of Japanese ancestry. Hum Biol.

[36] Hassanali J, Odhimbo JW (1981). Age of eruption of the permanent teeth in Kenyan African and Asian children. Ann Hum Biol.

[37] Shourie KL (1946). Eruption age of teeth in India. Indian J Med Res.

[38] Kaul S, Saini S, Saxena B (1975). Emergency of permanent teeth in school children in Chandigarh, India. Arch Oral Biol.

[39] Kaur B, Singh R (1992). Physical growth and age at eruption of deciduous and permanent teeth in well nourished Indian girls from birth to twenty years. Am J Hum Biol.

[40] Boesen P, Eriksen JH, Helm S (1976). Timing of permanent tooth emergence in two Greenland Eskimo populations. Community Dent Oral Epidemiol.

[41] Nystrom M, Kujala EK, Evalahti M, Peck L, Kataja M (2001). Emergence of permanent teeth and dental age in a series of Finns. Acta Odontol Scand.

[42] Folayan M, Owotade F, Adejuyigbe E, Sen S, Lawal B, Ndukwe K (2007). The timing of eruption of the primary dentition in Nigerian children. Am J Phys Anthopol.

[43] Feraru IV, Raducanu AM, Feraru SM, Herteliu C (2011). The sequence and chronology of the eruption of permanent canines and premolars in a group of Romanian children in Bucharest. OHMD.

